# A bibliometric analysis-based literature review of the relationship between sustainable water management and green innovations in the agricultural sector

**DOI:** 10.1016/j.heliyon.2024.e33364

**Published:** 2024-06-20

**Authors:** Anita Boros, Bianka Gordos, Dávid Tőzsér

**Affiliations:** a Institute of Agricultural and Food Economics, Hungarian University of Agriculture and Life Sciences (MATE), Páter Károly str. 1, Gödöllő, H-2100, Hungary; b Lajos Lőrincz Department of Administrative Law, Ludovika University of Public Service (NKE), Ludovika sq. 2, Budapest, H-1083, Hungary; c Doctoral School of Economic and Regional Sciences, Hungarian University of Agriculture and Life Sciences (MATE), Páter Károly str. 1, Gödöllő, H-2100, Hungary; d Department of Ecology, Faculty of Science and Technology, University of Debrecen, Egyetem sq. 1, Debrecen, H-4032, Hungary; e Circular Economy Analysis Center, Hungarian University of Agriculture and Life Sciences (MATE), Páter Károly str. 1, Gödöllő, H-2100, Hungary

**Keywords:** Green economy, Sustainability, Water management, Agroeconomy, Water resources

## Abstract

Global water demand has grown intensively over the last three decades, and the predictions suggest this trend will continue. Sustainable Water Management (SWM) defines water-based principles and action frameworks interconnecting societal, economic, and environmental aspects to establish and maintain good practices serving long-term objectives related to water resources. Water scarcity, deterioration of water quality, less effective water technologies, hydrological changes caused by climate change, and increased water demand require the thorough revision of conventional approaches, new methods, and new policy measures. The research methodology in this paper includes a comprehensive review and bibliometric analysis of relevant literature on water management and sustainable development, including empirical studies, theoretical frameworks, and policy documents. The study explores the conceptual context of SWM, reveals the barriers hindering its core progress, evaluates the impact of green innovations on the development of novel operations, and gets an insight into the current policy and regulatory framework for SWM. Besides giving a review of the current practices and perspectives in SWM, the results of this study contribute to a deeper understanding of the complex relationship between sustainable water management and green innovations in the agricultural sector and provide possible directions toward adopting effective strategies and policies to promote a more intense permeation of the SWM approach.

## Highlights

• The focus on Sustainable Water Management (SWM) has been increasing in the literature.
• Difficulties of various natures burden the in-depth spread of SWM.
• One of these is the inconsistent use and approach of the SWM definition.
• Green innovation is crucial in the widespread adaptation of SWM best practices.
• To succeed, societal, economic, and legislative change of perspective is required.

## 1 Introduction

Water plays a decisive role in ecosystems by substantially affecting their balance and development [1]. Considering that water has no substitute and has a finite quantity, the increasing water scarcity, which is acutely affecting extensive areas in Europe, warns us that the availability of this resource cannot be taken for granted, and persistent, effective, and systematic operations are required to support its protection [2].

The scientific community has drawn the vital role of water in the maintenance of life and ecosystems in many disciplines. The growth rate of freshwater demand is more than twice the population growth rate [[3], [4], [5]], while resource shortages often interrupt sustainability-related initiatives [6]. Globally, more than 1.5 billion people live in areas facing severe water-deficient conditions [7]. It is becoming increasingly evident that the preservation and distribution of this invaluable resource are essential not only for human survival but also for preserving the Earth's ecosystems and the well-being of future generations.

Various crisis indicators point to adverse global processes regarding water supply and quality. Among these, the literature highlights the inadequate protection of drinking water sources complemented by the obsolescence maintenance of existing infrastructure, the continuous growth of the global population, urbanization, climate change, and the gradual expansion of water-demanding (industrial) activities [8,9]. Water is identified as a fundamental factor in achieving sustainable development goals (SDGs) [10]. SDG 6.4 discloses that to handle the issue of water shortages by 2030, it requires a significant increase in the efficiency of water use in all sectors, as well as the establishment of sustainable water withdrawal procedures [11]. All this has led to the fact that the World Economic Forum named the water crisis one of the five most significant global risks regarding impacts threatening society [12].

It was previously recognized that agriculture consumes the most water among sectors, and it has one of the highest contributions to water crises, especially in semi-(arid) regions [13,14]. It also means, at the same time, that agriculture bears the highest potential to significantly contribute to a sustainable transition in economic, societal, and environmental management practices on a global scale. In agriculture, several parameters influence water use and management, out of which localization and scheduling of irrigation are two of the most decisive factors [15]. However, the implementation and introduction of efficient, state-of-the-art methods are often aggravated by high costs, reliability shortcomings, and developmental process problems [16]. Based on these previous, overcoming the potential and actual barriers, thereby supporting the recent and future needs of all the sectors, a systematic approach should be among the key points of sustainable water management [17].

This study uses bibliometric analysis and provides a concise literature overview to assess the current issues and trends of water management, especially the relationships and definitions of SWM and agriculture. During our research, we were looking for an answer to what green innovations support the water management processes in the sustainable transition. One of the basic assumptions of this paper is that the lack of a uniform definition of SWM in the studied literature makes it difficult to develop and employ adequate methods and techniques. It is also assumed that although agriculture and SWM are at the forefront of research, the number of studies on agricultural water management through green innovations is still negligible. This presupposes the elaboration of new adaptive water policies – considered best practices – worldwide and their representation in the literature.

## 2 Literature analysis of SWM-related publications

An extensive literature scan was conducted in the Web of Science (WoS) database using topic search terms to test the assumptions of this paper. Various term combinations have been tested to assess the number of potential matches. At first, a search was run using the term "sustainable water management” for the documents published between January 01, 2005 and July 01, 2023, which resulted in more than 30,000 matches. Due to this numerosity of relevant publications, search parameters were refined; the terms "sustainable water management” AND "definition” were applied, providing 15 matches. Therefore, applying the terms "sustainable water management" AND " innovation" was performed, whereby 18 matches were listed. Among the studies found within the two latter searches, the main topics of most papers were connected to water economics, followed by environmental sciences and sustainable green technologies. Additionally, the terms "water management" AND " innovation" were surveyed, resulting in 421 matches. These main topics were environmental sciences, water economics, and sustainable green technologies. This latter set of listed publications was analyzed in R using the *bibliometrix* package [18].

**Fig. 1:**
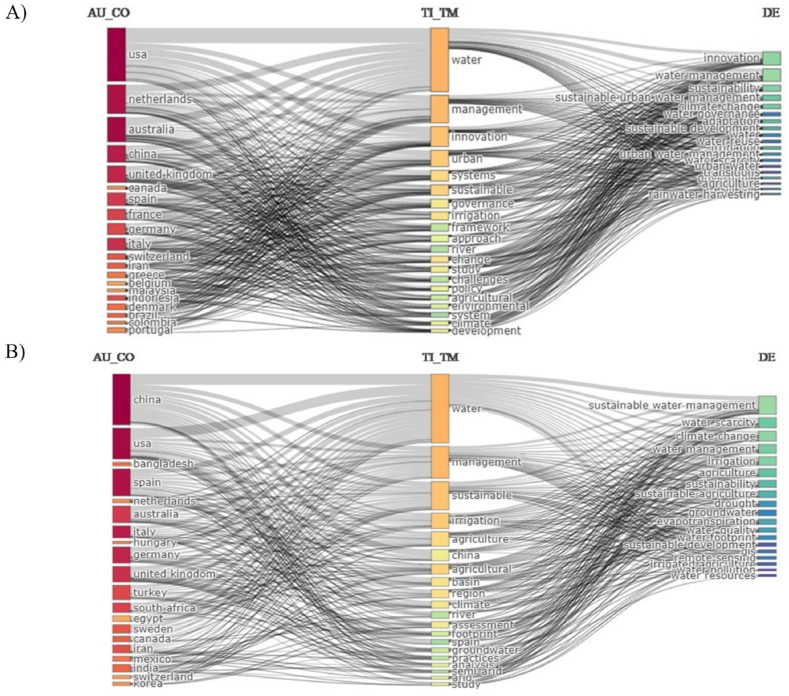
Distribution of publications by geographical area, topic, and keyword listed after applying the search terms "water management" AND "innovation" (A) and "sustainable water management" AND "agriculture" (B). Source of data: Web of Science, self-edit.

The cited documents were published by 1562 authors; most were journal articles (340), while the remaining publications were books, book chapters, reviews, or proceedings papers. The first 20 topics with the most matches in the water management-innovation relation could be linked to researchers from the US, the Netherlands, Australia, China, and the United Kingdom. It can also be seen in Fig. 1A, which focus areas appeared in the titles of the studies most frequently; in line with the search terms, the three most mentioned areas are water, management, and innovation. The keywords referred to in the publications showed a similar picture; innovation, water management, and sustainability are the most used ones. On the other hand, evaluating sustainable water management from the agricultural perspective, authors from China, the USA, and Spain prepared relevant papers the most frequently (Fig. 1B). Among the focus areas, the most common terms were water, management, and sustainable in the titles of publications. Fig. 1B also shows that sustainable water management, water scarcity, and climate change were the most used by the distribution of keywords. It is presumed that afore differences in geographical scientometrics patterns are widely associated to the economical preferences, opportunities, and challenges of individual states. However, some regions with exceptional risk of agricultural water scarcity and supply issues are highly underrepresented in the demonstration above. In Africa, for instance, dozens of states are burdened with quantity and quality-related deficiencies of available water, which risks are, in most cases, introduced in details by local researchers with tight resources in journal with currently lower impact, while these problems are assessed barely or in a generalized way by authors with better accessibility to scientifically more recognized platforms [19].

**Fig. 2:**
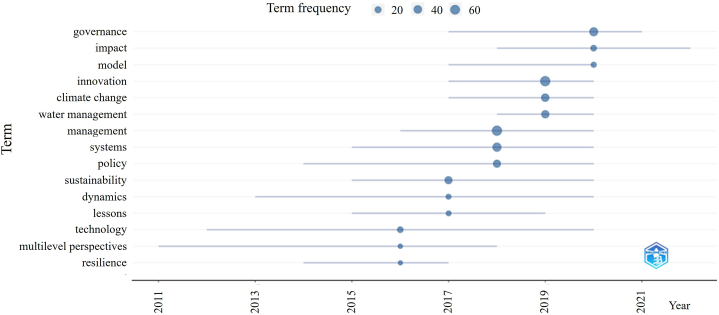
Evolution of research trends for publications listed after applying the search terms "water management" AND "innovation". Source of data: Web of Science, self-edit.

It was also highlighted that the focus of scientific interest on management-like issues has shifted towards innovation, governmental, and administrative issues in recent years (Fig. 2). The intense distribution of afore terms can be related to the trends to support the simultaneous involvement of environmental, social, and governmental aspects on multiple scales, therefore, its manifestation in the agriculture-related scientific literature is considered a feedback regarding the extensive penetration of ESG approach. Additionally, due to the relative low number of publications in the studied topics, factors like publishing papers on actual hot topics and the launch of special issues around specific segments could widely determine the frequency and composition of highlighted terms along the studied timespan.

Further, after modifying the filter parameters to "sustainable water management" AND "agriculture", 155 publications were resulted in the WoS database with nearly 700 authors. The annual growth rate of publications in this area is 10.45%. Most papers were published in *Water*, followed by *Sustainability* and *Science of the Total Environment* (Table 1). It is also noticeable that based on their metrics, the most relevant journals belong to the top fraction of the ones having publications in the related topics. It is worth noticing that there are thousands of journals that are not indexed in the Web of Science (Core Collection), therefore, these were not included in this synthesis. According to the findings above regarding the publication opportunities of authors from states with increased concern of agricultural water supply issues and based on the relative low number published papers presented in Table 1, the inclusion of each journal article available online would presumably alter the proposed list of top 10 journals.

**Table 1:** The list and major scientometric parameters of 10 journals publishing the most papers with the search terms "sustainable water management" AND "agriculture". Ranking: the highest Q-ranking of the journal and its prevalence (in parentheses) in the last 10 years (between 2014 and 2023).

Journal	Nr. of papers published	Citations per document (2 years, 2023)	Ranking (2014–2023)
Water	13	3.274	Q1 (2)
Sustainability	9	3.953	Q1 (4)
Science of the Total Environment	8	9.439	Q1 (10)
Agricultural Water Management	7	6.878	Q1 (10)
Journal of Environmental Management	6	9.317	Q1 (10)
Journal of Cleaner Production	5	11.084	Q1 (10)
Land Use Policy	5	6.944	Q1 (10)
Sustainable Water Resources Management	4	2.392	Q2 (3)
Irrigation and Drainage	3	1.775	Q2 (6)
Water Policy	3	1.642	Q2 (10)

**Fig. 3:**
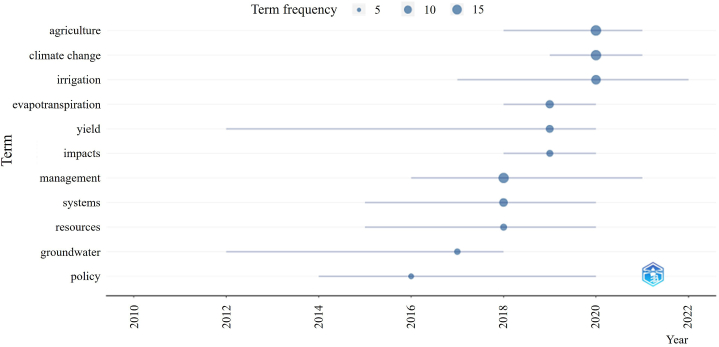
Evolution of research trends of publications listed after applying the search terms "sustainable water management" AND "agriculture". Source of data: Web of Science, self-edit.

In terms of agricultural aspects of SWM, distinct trends can also be recognized. Fig. 3 shows that the former management focus has shifted in recent years towards dealing with climate change issues, including the prioritization of irrigation problems. The prominence of these afore terms are supported by the fact that climate change-related irrigation problems are getting increased attention due to the very extensive scope of concerned parties ranging from producers to consumers, also including legal bodies and researchers. Besides being highlighted on multiple levels, there are still some major factors hindering the efficiency of climate adaptation: questionnaire surveys shed light on the fact that there are significant differences in individual attitude regarding such practices, which has to be formed in order to support higher-scale initiatives [20].

### 2.1 The definition of sustainable water management

According to the UN Report on Sustainable Development Goals from 2021, two billion people still did not get safely managed drinking water in 2020, 3.6 billion people did not have managed hygiene facilities, and 2.3 billion people's fundamental hygiene conditions were still not provided. Further, by the progress taken to that date, 129 countries were not even close to reaching sustainable water resources by 2030. Water scarcity, defined as an imbalance of water demand and supply, is a great challenge [21]. In Resolution 64/292, the UN General Assembly acknowledged the right to safe and clean drinking water and sanitation as a human right that is essential for the full enjoyment of life and all human rights. In another aspect, water scarcity and deterioration of water quality significantly affect soil properties, food production, and nutrition for all organisms. Irrigation water in agriculture accounts for 70% of global freshwater consumption [22] and is strongly related to food safety, economic development, and environmental impacts [23,24], which affect the agricultural production and sustainable development [25]. However, these afore segments cannot be handled separately; the demand and supply risks require a complex approach, which is not achieved in many instances, therefore, effective transition to a more sustainable water management is often hindered [26]. Groundwater stocks account for nearly one-third of the world's withdrawn and utilized water resources; thus, protecting these supplies is considered a prerequisite for the adoption and enforcement of rights intended by the UN [27].

As can be seen, the concept of SWM arises at international levels. However, its terminology has not fully evolved yet. A study by Delgado-Serrano and Borrego-Martin [28] emphasized that SWM is dedicated to addressing water management, water supply, and water quality problems by implementing sustainable solutions, including the effective and reasonable treatment of groundwater. At the same time, many authors underline that the actual emergence of the concept of SWM is also hindered by the lack of a commonly approved definition of sustainability, which may – among others – result in the failure of cooperation between individual sectors and can make the adoption of practices more difficult [29,30]. Recognizing that the conceptualization has basic importance, it would also be important to provide, besides general findings, tailored recommendations to states that have limited availability of management tools; developing countries having immense-scale agricultural water crisis should be supported on each level of operation [31].

The consolidated approach to understanding the complexity of sustainability problems and solutions is represented by research on socio-ecological systems (SES) [32]. SES are systems formed by the complex interaction of nature, economy, and society, which are the core research units of scientific sustainability evaluations [33]. The framework of SES was used to understand and manage agricultural and water sustainability problems from the economy to the global size [[34], [35], [36]]. In this regard, other parameters and continuous adaptation to recent challenges must also be focused; sociocultural factors are usually less addressed ones during these evaluations, despite the fact that these have potential to alter people's attitude toward a more conscious approach in water management [19].

The Food and Agriculture Organization of the United Nations (FAO) defines the sustainability of agricultural water use as sustainable water use balances the social, human, economic, and environmental demands for good quality freshwater (and the benefits of those uses) with the long-term availability of utilizable and replenishable surface and groundwater resources. It seeks to do this at minimum economic, social, and environmental costs [37]. The World Bank [38] defines sustainability as better water management, representing great economic, environmental and social benefits. The International Commission on Irrigation and Drainage (ICID) defines sustainability as the management of a watershed system with sustainable technological options, which may ensure the sustainability of the land, agriculture, and forestry or its combinations to conserve natural resources, with adequate institutional and economical options [39]. As proposed earlier, creating the definition not implicitly gives the frames of sustainable water management in the individual communities or states, however, in the absence of such conceptualization major and effective operation cannot be launched in favor of a more climate-resilient agriculture.

This paper also assessed publications found in WOS based on the "sustainable water management" AND "definition” search terms to evaluate the elements of SWM. This mainly aimed to find out if the listed publications determine the definition of SWM. As presented in Table 2, most papers did not give exact definitions of how they interpret the concept but instead sought to describe the frontiers or related definitions.

**Table 2:** The determination of the definition of SWM in the publications in the literature.

Publication	Does the publication determine the definition of sustainable water management? In what aspects?	Further related definitions included
Sheng et al. [40]	No	Green infrastructure
Dahal et al. [41]	Yes; ensuring quality with minimal adverse effects, stakeholder satisfaction, well-being, equal participation in processes, promotion of long-term economic growth, extensive consideration of climate-related issues.	
Richter [42]	Yes; management of water quality and withdrawal, supporting long-term needs, joint decision and action from stakeholders against excessive and adverse human influence.	
Folke [32]	Yes; complex approach of sustainability-related issues based on socio-ecological systems (SES), supported by scientific research.	
Lescourret et al. [34]		
Moraine et al. [35]		
Piemontese et al. [36]		
Castelli et al. [43]		
Newig and Challies [44]	No	Integrated Water Resources Management (IWRM).
van der Laan et al. [45]	No	Sustainable land management
Tarolli and Straffelini [46]	No	Sustainable agriculture
Wang et al. [47]		
Novoa et al. [48]		

Based on the above literature findings and the results of this bibliometric analysis, the elements of SWM can be determined as follows:

Management of water resources based on the natural characteristics of the water cycle and diverse local water sources in a specific way using a multidisciplinary, integrated approach, which aims to

- promote the calculable water withdrawal in favor of water resources management, improvement of water quality, and water saving, and apply alternative solutions to support the water cycle and improve the efficiency of rationalized water use;
- enable the enforcement of water-related benefits to overcome the natural risks of social and economic challenges with appropriate and proportionate solutions, thus interconnecting the requirements of all segments of sustainability to minimize the adverse natural effects of water management, thereby securing societal participation, human well-being, biodiversity, and economic growth;
- change water management policies in sectors (e.g., agriculture) being highly dependent on water resources to achieve SWM according to the most efficient solutions achievable. Russo et al. [49] emphasized that SWM requires the rational distribution between competing water sector needs and the balance of financial and social resources needed to support the fundamental water systems. Marlow et al. [50] underlined the importance of water cycle-based policies.

The value of these afore components of SWM in considered a major step in supporting regional and global goals. On the other hand, it seems clear that the realization of these measures in the form of presentation of best practices and case studies would also aid the success of implementation for further states facing relevant challenges. Therefore, focusing on the scientific literature introducing the detailed circumstances of execution and environmental, social, and economical consequences in key.

### 2.2 Difficulties hindering the implementation of sustainable water management

Publications on sustainability issues of water management focus primarily on environmental sustainability, followed by the economic and social dimensions [51,52]. Table 3 presents several papers and their core findings on the difficulties of SWM's measurement and quality assurance.

**Table 3:** The determination of difficulties related to SWM in the studied literature.

The nature of the difficulty (main division)	Difficulties within the main divisions	Papers addressing the difficulties
Difficulties arising from the specifics of water management systems	The size of water management systems (e.g., watershed),	Chen et al. [53]
	Changing meteorological conditions,	Dong et al. [54]
	Transnational nature of water systems,	Jetoo [55]
		Schmidt [56]
	Irreplaceability of water,	Gouvea et al. [57]
	Fragmentation of the global water market,	O'Callaghan et al. [58]
Technological difficulties	The measurement and analysis problems of these technologies hinder the spread of new technologies,	Garcia et al. [59]
	Water infrastructures based on outdated technologies,	Sahoo and Goswami [60]
	Inadequate maintenance of water infrastructures,	Taiwo et al. [61]
Innovation difficulties	Distrust for novelties,	Ramos [62]
	Water management has still been an overly complex and fragmented area, which relies on conventional technical and linear economic approaches,	Brown and Farrelly [63]
		Wan Rosely and Voulvoulis [64]
	The spread and introduction of new technologies are slow due to the underdevelopment of competencies related to technical, environmental, societal, and economic competitive goals,	Thomas and Ford [65]
		Wehn and Montalvo [66]
		Yigezu et al. [67]
	The long replacement cycle of technologies,	O'Callaghan et al. [58]
Policy difficulties	The societal, ecological, and technical complexity of issues present massive challenges to policymakers during the design of water management plans at the municipality and state levels,	van der Leer et al. [68]
	Lack of uniform and universal water management regulatory policies,	Bolognesi [69]
	Inadequate quality assurance systems,	Refsgaard et al. [70]
		Babiso et al. [71]
	Disproportionate water pricing,	Rogers et al. [72]
		Zhang and Oki [73]
	Incomprehensive control by authorities,	Cosgrove and Loucks [74]
	Lack of professionals having adequate expertise to integrate SWM and green innovation solutions,	Dehnavi and Al-Saidi [75]
Societal difficulties	Lack of relevant education for users,	Garcia et al. [59]
	Lack of understanding regarding the contribution of water to the achievement of sustainable development goals,	Guppy et al. [76]
		di Vaio et al. [77]
	I. Evaluation of 53 studies and the analysis of 12 barrier types; difficulties are mostly socio-institutional, with only sporadic strategies against them. The solution would be complex transition programs	Brown and Farrelly [63]
	II. Evaluation of 31 barriers in six types, widely overlapping with the previous classification.	Vasconcenlos et al. [78]

As can be seen, the difficulties presented in the scientific literature are of various nature. It suggests that the management of agricultural water-related issues should be considered in a complex vision. At the same time, it should also be kept in mind that complexity-based overgeneralization is a phenomenon to be avoided; rather, to maintain well-founded information, in-depth analyses are needed for each difficulty presented in Table 3, complemented by the persistent revision of novel ones based on a fluent communication between the scientific community and all the parties concerned by agricultural water management.

### 2.3 The role of green innovation in sustainable water management

As can be seen above, it was presumed and found in this paper that the lack of a uniform definition of SWM in the literature hinder the development and execution of adequate methods and techniques. It was also hypothesized that number of publications that serve to develop agricultural water management through green innovations is still low. To frame the concept, green innovation means the development of environmentally friendly products and processes [79], which can help reduce environmental stress and mitigate natural deterioration at all stages [80]. For the agricultural sector, literature sources emphasize that agriculture, as the most water-dependent economic sector, must increase its efforts to comply with recent challenges [81,82]. In solving this issue, green innovations can play an essential role in the transition to sustainable technologies [83]. As presented earlier, several difficulties have been uncovered and addressed by researchers in the field. Many of these papers agree that the success of sustainable transition cannot be granted without adopting modern and state-of-the-art technologies, however, presentation of practices, benefits, and results of actual implementation of such innovations are still scarce. Therefore, complementary operations are needed.

As one of the major segments, ecological modernization focuses on policies that improve water efficiency and treatment through technological innovation and changing individual behavior [84]. During the associated green innovations, high investments, risks, and extended research and development cycles are required. Therefore, businesses must face significant financial and non-financial difficulties in implementing innovative measures [85]. Further, the lack of a widely accepted classification system and lacking precise innovation terminology often hinder the development and acceptance of green technology [58,86]. Ekins [87], for example, emphasizes that it requires a technological transition with strong government and regulatory provisions if eco-innovation seeks to initiate a sufficiently high-scale technological transition. In relation to this previous, there exists a huge gap among states in the receptivity of novel technologies, which pattern is noted also in the scientific literature [88]. The studied research papers reveal this; however, the emphasis is put on a descriptive approach rather than (stepwise) recommendations for the catching up of – in this regard – underprivileged economies.

Many authors agree that green innovation can be a breakthrough in developing sustainability in agricultural production [89]. Porter and van der Linde [90] found that strict but adequately designed environmental regulations could trigger innovation and enhance competitiveness. Tong et al. [91] pointed out that environmental regulations can encourage businesses to invest in green technologies, improving companies' innovation ability. Klaassen et al. [92] also emphasize that the level of development of green innovation varies depending on the rigor of the environmental regulation of countries. To amplify the importance of legal aspects in sustainable agricultural water management, attention is dedicated in this study to the role of policies.

### 2.4 Sustainability approach in water policy

According to estimates, by 2050, due to rapid population and economic growth, declining water resources, and globally increased pollution, nearly 6 billion people are expected to suffer from water scarcity [93]. Therefore, developing proportionate water management policies and strategies is essential for long-term sustainable water management. States with various water policy regulators often define residential and economic water-use rules. Many states have water supply problems, as demand is significantly higher than the amount of water that can be used in water management on a sustainable basis. Water utilities often operate based on inadequate business models, generally with too small supply areas and poor infrastructure [94]. To take control of the negative processes, for instance, Portugal, Italy, and Spain have introduced various tax systems for agricultural water withdrawal. France, Portugal, and Italy have already employed withdrawal taxes on all water sources (surface- and groundwater) for water-saving purposes, while in Denmark, the "eco-tax" on pesticides and agricultural chemicals supports the maintenance of water quality [95]. In developed economies (e.g., OECD countries), such taxes already have proven force to trigger the use of renewable resources and lower the mean ecological footprint of the concerned states, however, yet to a certain extent that can and should be further increased [96].

One of the most significant problems is that the water-related issues are usually treated in various scales among states. This phenomenon results in fragmented regulators, different technological solutions, and water management practices without a cross-border approach. In recent years, the scientific community has worked intensively to propose solutions and alternatives to public utilities to improve their operation and coordination of water management [[97], [98], [99], [100]]. As assumed in the hypotheses here, most authors highlight transnational and systemic solutions by introducing adaptive systems that minimize risk and reduce vulnerability [97,101]. According to the previous literature and the authors of this paper, for adaptive management and governance, the following actions and challenges should be considered.

- Cooperation-based governance should be preferred in water management [102],
- Questions for environmental flexibility are context-dependent, incidental, and depend on many different stakeholder groups and governmental scales [101],
- Sustainable practices are of paramount importance in the planning and operating phases of water systems [103],
- Innovation is considered the main driving force of sustainable transition [30,104,105],
- Legal frameworks supporting water management are essential for national policy planning at the national level [73],
- Some authors have also elaborated sustainability indices for water resources planning and management [106], while many design, optimization, and GIS methods have also been developed in recent years to support SWM [[107], [108], [109], [110], [111], [112]],
- Ashwini et al. [113] presented that introducing modern irrigation systems has reduced water withdrawal by 50%, which did not significantly improve water saving. The authors demonstrated that changing the water-demanding sowing patterns has effectively improved the sustainability of blue water and groundwater by regulating the bounce effects,
- Mintrom and Rogers [105] named six key measures [i) Clarify the problem and articulate a clear vision; ii) engage others to identify workable solutions and implementation pathways; iii) secure support from influential stakeholders; iv) establish practical monitoring tools and learning systems; v) foster long-term relationships of trust and mutual support; vi) develop narratives that support on-going action] to promote sustainability transitions,
- According to Garcia et al. [59], integration of Intelligent Network Systems, Digital Twins, Big Data analytics, remote sensing, and various information and communication technologies is essential to reduce the risks related to flow, pressure, capacity distribution, and users. Additionally, many authors apply various model-controlled regulatory strategies [114]. It was also underlined that computer simulation models can be used to explore and compare the results and compromises related to actual and drafted long-term water maintenance solutions [115],
- In 2006, the United Nations (UN) proposed the introduction of the Integrated Water Resources Management (IWRM), which approves and confirms the management framework so the decision-making process can handle current water-based challenges [116].

As the publications above are parts of the sample evaluated in the literature analysis and the policy relations appear frequently according to the analysis of the keyword frequency, it can be stated that the scientific community dedicates much attention to introducing policy creation and implementation in the agricultural water management. However, based on the specific core topics of papers, the level of representation and elaboration need further improvement. Knowing that the evolution of legal frameworks is still in progress by many governments in response to the climate-related stress and global initiatives and directives, a more thorough penetration is expected in the next few years, resulting in more publications and the actual presentation of achievements of short- and mid-term progress.

## 3 Conclusions

Water management is a fundamental set of actions supporting the water supply, focusing on fulfilling quality and quantity parameters. Recently, this conventional approach, however, needs to be revised and reconsidered to keep track of the economic, societal, and environmental processes that exploit water resources to a degree that – assuming intensifying or even constant rate of use – cannot be maintained without the emergence of significant harms in each of the afore systems. These justify the need to transition water-related practices toward sustainable water management. As introduced in this paper, the issue of fair water practices has been gaining more and more attention in recent years, proven by the core topics of relevant publications. It was also revealed that elaborating the definition of SWM has become prominent from slightly different aspects. It was also demonstrated that SWM adaptation is burdened by difficulties of various natures, making its realization slow and circumstantial. To trigger the sustainable transition, green innovations play a primary role in accelerating and making progress more efficient by introducing state-of-the-art technologies. Modern operations in policymaking can also aid desired perspectives, with now only a sporadic occurrence of best practices worldwide. An integrated approach in water management executed on the most reasonable administrative level and the adoption of proven best practices by states and areas with much space for relevant development can be a significant step forward in favor of the distribution of SWM practices on a global scale. The authors of this paper believe that the first step of this phenomenon should be a change of attitude of all parties involved, including – among others – leading representatives, legislative bodies, suppliers, and users.

## Funding

This project was supported by the Doctoral School of Economic and Regional Sciences of the Hungarian University of Agriculture and Life Sciences (MATE; general support) and the NRDI (National Research, Development and Innovation Office) Fund (grant number 138806).

## Data availability statement

Data associated with this bibliometric analysis-based literature review have not been deposited in any public repository. All data used in this article have been referenced herein.

## CRediT authorship contribution statement

**Anita Boros:** Writing – review & editing, Writing – original draft, Supervision, Resources, Project administration, Methodology, Investigation, Funding acquisition, Conceptualization. **Bianka Gordos:** Writing – review & editing, Writing – original draft, Methodology, Formal analysis. **Dávid Tőzsér:** Writing – review & editing, Writing – original draft, Visualization, Resources, Methodology, Formal analysis.

## Declaration of competing interest

The authors declare that they have no known competing financial interests or personal relationships that could have appeared to influence the work reported in this paper.
